# Higher fracture risk associates with worse cognitive performance in Mexico

**DOI:** 10.1002/alz.095801

**Published:** 2025-01-09

**Authors:** Karla Paulina Ruiz‐Castillo, Natalia Pozo Castro, Regina Silva Paradela, Victor Valcour

**Affiliations:** ^1^ National Institute of Neurology and Neurosurgery Manuel Velasco Suarez / Mexican Social Security Institute, Torreón, CU Mexico; ^2^ Global Brain Health Institute, San Francisco, CA USA; ^3^ Hospital San Borja Arriarán, Santiago, Region Metropolitana Chile; ^4^ Global Brain Health Institute, Memory and Aging Center, University of California San Francisco, San Francisco, CA USA; ^5^ University of Chile, Santiago, Region Matropolitana Chile; ^6^ University of São Paulo Medical School, São Paulo, São Paulo Brazil; ^7^ Memory and Aging Center, Weill Institute for Neurosciences, University of California, San Francisco, San Francisco, CA USA; ^8^ Global Brain Health Institute, University of California, San Francisco, San Francisco, CA USA

## Abstract

**Background:**

Osteoporosis is associated with an increased risk of dementia (Zhang, P. et al., 2022). The Fracture Risk Assessment Tool (FRAX) calculates the risk of hip fracture using a series of clinical variables, even in the absence of bone density knowledge. A higher FRAX scores indicates higher risk for osteoporosis. Some studies suggest that individuals with higher FRAX are also at increased risk of cognitive decline and dementia, with less evidence from low‐ and middle‐income countries (LMIC). We aim to evaluate the association between FRAX score and cognitive impairment in Mexican older adults.

**Method:**

This is a cross‐sectional study of 500 Adults 60 years of age or older enrolled at a dementia clinic in a tertiary care hospital in México who completed baseline evaluations. We assessed the risk of hip fracture using FRAX and cognitive performance with the MMSE. Participants also received brain MRI (1.5 tesla), and a comprehensive geriatric assessment. A linear regression was performed to evaluate the association between FRAX score and cognitive impairment measured through MMSE.

**Result:**

The sample included 295 (59%) women and 205 men (41%), with a mean age of 69±6.4 years, and a mean of 6±4.7 years of education. We excluded 1.8% of cased due to probable delirium. Among the remaining cases, 97.7% were cognitively impaired, which was classified as: mild cognitive impairment (MMSE between 24‐29, 40.8%) and dementia (MMSE < 24, 59.2%). The most prevalent diseases were hypertension (61.8%), diabetes (35.8%), and depression (23.2%). We found a negative association between the FRAX score and the MMSE score (*b* = ‐0.288, *p* = 0.001), after controlling for age, sex, and education.

**Conclusion:**

A higher FRAX score was associated with a lower MMSE score in Mexican older adults, supporting an association between fracture risk and cognitive impairment. Understanding this association is important for developing integrated strategies for the prevention and management of both osteoporosis and dementia in aging populations from LMIC. Further research is warranted to elucidate the underlying mechanisms linking osteoporosis to dementia.

